# The multiple sclerosis relapse experience: patient-reported outcomes from the North American Research Committee on Multiple Sclerosis (NARCOMS) Registry

**DOI:** 10.1186/1471-2377-13-119

**Published:** 2013-09-10

**Authors:** Molly Nickerson, Ruth Ann Marrie

**Affiliations:** 1 Questcor Pharmaceuticals, Inc, 26118 Research Road, Hayward, CA 94545, USA; 2 University of Manitoba, Winnipeg, Canada, Health Sciences Centre, GF543-820 Sherbrook Street, Winnipeg, Manitoba R3A 1R9, Canada

**Keywords:** Multiple sclerosis, Relapses, Corticosteroids, Methylprednisolone, Prednisone

## Abstract

**Background:**

Among patients with relapsing-remitting multiple sclerosis, relapses are associated with increased disability and decreased quality of life. Relapses are commonly treated with corticosteroids or left untreated. We aimed to better understand patient perceptions of the adequacy of corticosteroids in resolving relapse symptoms.

**Methods:**

We examined self-reported data from 4482 participants in the North American Research Committee on Multiple Sclerosis (NARCOMS) Registry regarding evaluation, treatment, and recovery from relapses. Pearson’s chi-square test was used to analyze categorical variables, while logistic regression was used to assess factors associated with patients’ perceptions.

**Results:**

Forty percent (1775/4482) of respondents were simply observed for disease worsening, whereas 25% (1133/4482) were treated with intravenous methylprednisolone (IVMP) and 20% (923/4482) with oral corticosteroids; additional treatments included adrenocorticotropic hormone, plasmapheresis, intravenous immunoglobulin, and others. Among patients who responded to questions about their most recent relapse, 32% (363/1123) of IVMP-treated and 34% (301/895) of oral corticosteroid-treated patients indicated their symptoms were worse one month after treatment than pre-relapse, as did 39% (612/1574) of observation-only patients; 30% (335/1122) of IVMP-treated patients indicated their treatment made relapse symptoms worse (13% [145/1122]) or had no effect (17% [190/1122]), as did 38% (340/894) of oral corticosteroid-treated patients (worse, 13% [116/894]; no effect, 25% [224/894]) and 76% (1162/1514) of observation-only patients (worse, 17% [264/1514]; no change, 59% [898/1514]).

**Conclusions:**

Overall, patients with relapsing multiple sclerosis who receive treatment report better outcomes than those who are simply observed. However, a sizeable percentage of patients feel that their symptoms following corticosteroid treatment are worse than pre-relapse symptoms and that treatment had no effect or worsened symptoms. Patient perceptions of relapse treatment deserve more attention, and more effective treatment options are needed.

## Background

Relapses are the hallmark of multiple sclerosis (MS), with approximately 80% of MS cases diagnosed as relapsing-remitting (RRMS) at onset [1,2]. Symptoms of relapses vary and commonly include sensory disturbances, fatigue, and/or motor impairment; symptoms may last from several days to several weeks and generally resolve over time, with or without treatment, but recovery may be incomplete [1]. A substantial proportion (up to 49%) of RRMS patients exhibit residual deficits on the Expanded Disability Status Scale (EDSS) after relapses, including patients who received treatment [3,4]. Further, relapses have substantial emotional and psychosocial effects [5,6] and significantly increase economic costs, including substantial indirect costs associated with reduced productivity and declines in functional ability [7].

Although mild relapses may be managed with observation only, severe symptoms and significant functional limitation generally prompt treatment with high-dose corticosteroids [8]. Such treatment shortens the time to recovery from a relapse [9]; however, evidence that this improves the extent of recovery is lacking, and corticosteroids are not tolerated by all patients. Adverse effects of acute treatment with high-dose corticosteroids may include gastrointestinal disturbances, headache, increased appetite, hyperglycemia, edema, effects on mood, anxiety, insomnia, and, rarely, psychosis; avascular necrosis of bone or hypokalemia are also possible but occur infrequently [8].

Patient-reported outcomes, including health-related quality-of-life (HRQoL) measures, are increasingly attracting interest within MS research. Patient-reported HRQoL may predict disability in MS [10] and is associated with magnetic resonance imaging (MRI) lesion burden and brain atrophy [11]. However, patients’ perceptions of recovery from relapses, specifically with regard to the effects of treatments, have not received as much attention. Further, patients’ perceptions of improvement do not necessarily correlate with clinical measures. For example, one study found that, 6 weeks after treatment with intravenous methylprednisolone (IVMP), 9% to 23% of patients had significant improvement on clinician-rated measures, but on patient self-report, only 5% indicated a complete return to baseline [12].

Our goal was to quantify the subjective patient experience following treatment with corticosteroids, via a retrospective analysis of data from the North American Research Committee on Multiple Sclerosis (NARCOMS) Registry. We also investigated the relationship between patients’ characteristics and perceived relapse outcomes.

## Methods

### Registry and participants

The NARCOMS Registry is a long-term project of the non-profit Consortium of Multiple Sclerosis Centers (CMSC) [13,14], designed to promote MS research. Patient participation in NARCOMS is voluntary. NARCOMS was founded by the CMSC in 1993 and, over the years, has been supported by grants and in-kind services from the United Spinal Association, the Paralyzed Veterans Association, and the National Multiple Sclerosis Society, and by unrestricted grants from Berlex, Biogen Idec, Immunex, EMD Serono, and Teva Neuroscience. It has registered more than 36,000 participants to date [15]. Registry participants are asked to complete 2 update questionnaires annually to provide current demographic and clinical information. The NARCOMS Registry protocol is approved by the Institutional Review Board at the University of Alabama at Birmingham. Participants give written consent for their information to be used for research purposes.

Access to the limited de-identified dataset was obtained through a research agreement between the Consortium of MS Centers (CMSC)/NARCOMS and Questcor Pharmaceuticals. The NARCOMS registry operates under the oversight of the Institutional Review Board at University of Alabama at Birmingham.

### Outcome measures

This analysis included data derived from the spring of 2007 update questionnaire, which included questions pertaining to relapse management, disease-modifying therapies (DMTs), and symptomatic therapies. Participants identified the type of treatment they received for their most recent relapse (options were: observation only, IVMP, IV dexamethasone, oral MP, oral dexamethasone, oral prednisone, intramuscular [IM] adrenocorticotropin hormone [ACTH], IV immunoglobulin [IG], plasmapheresis, and other) and were asked to evaluate their response to treatment. Only those responders who reported having experienced a relapse were included in this study; for questions regarding the most recent relapse, there was no restriction regarding the time frame when that relapse occurred.

Two specific questions were explored in detail for this analysis. The first was used to evaluate patients’ perceptions of recovery in terms of *symptom improvement or resolution*: “As compared to the symptoms just before my most recent relapse, my overall MS symptoms 1 month after the relapse treatment were…” The second was used to evaluate patients’ perceptions of the *effect of treatment* on facilitating or improving recovery: “As a result of the treatment, my recovery was…” Response choices for both questions were on a 7-item Likert-type scale: much worse, worse, a little worse, no change, a little better, better, or much better.

### Statistical analysis

Pearson’s chi-square test was used to compare the distributions of categorical variables and to test for statistical independence between them. Logistic regression was used to assess factors associated with patients’ perceptions of treatment efficacy using all participants who responded to relevant survey questions. For this analysis, the 7 response choices were collapsed into 2 categories for each question. For the question on symptoms, responses were categorized as worse (responses = much worse, worse, a little worse) or not worse (responses = no change, a little better, better, much better); for the question on treatment, responses were categorized as not better (responses = much worse, worse, a little worse, no change) or better (responses = a little better, better, much better). The factors evaluated were sex (male vs. female), number of total lifetime relapses (1–4, 5–9, or ≥10 [i.e., based on tertiles]), time since most recent relapse (months [as a continuous variable]), treatment (IV corticosteroids, oral corticosteroids, other treatment [IM ACTH, IVIG, plasmapheresis, unspecified], or observation), and treatment location (home/nonclinical site, clinical site, or unspecified site). Prior to the fitting of logistic regression models, the distributions of each categorical factor were reviewed in order to confirm that the sample size for each level was sufficiently large. In addition, the distributions of each quantitative factor were reviewed to ensure that there were no extreme outliers. The initial logistic regression model for each of the two endpoints of interest included main effects for each of the above factors. Based on the use of individual and joint tests of the effects of selected factors, a reduced model identified factors significantly associated with patient-reported symptom resolution and treatment effects. Statistical significance was set at *p *< 0.05. SAS version 9.1 was used for all analyses (SAS Institute Inc., Cary, N.C.).

## Results

### Demographic and clinical characteristics

A total of 10,688 respondents (of 16,590 enrollees active at the time) completed the spring 2007 update survey [16]. The study population for this analysis included 4482 respondents who reported having experienced a relapse in 2004, 2005, 2006, or 2007 (Table 1).

**Table 1:** Demographic and clinical characteristics of respondents

**Characteristic**	
Age, years (n = 4479)	n (%)
20-24	8 (<1)
25-34	277 (6)
35-44	1015 (23)
45-54	1795 (40)
55-64	1127 (25)
65-74	214 (5)
75-90	43 (1)
Sex (n = 4482)	n (%)
Male	854 (19)
Female	3628 (81)
Number of lifetime relapses (n = 4442)	n (%)
1-4	1420 (32)
5-9	1466 (33)
≥10	1556 (35)
Months since last relapse	Mean (SD)
Overall (n = 4367)	11.7 (9.8)
Patients with 1–4 relapses (n = 1389)	14 (10.8)
Patients with 5–9 relapses (n = 1440)	12 (9.4)
Patients with ≥10 relapses (n = 1502)	10 (8.7)

### Relapse assessments, management, settings, and follow up

Prior to the decision regarding treatment, approximately 62% of relapsing patients were examined by a physician and 18% by a physician’s assistant or nurse, 36% consulted with a health care provider by phone, 25% had an MRI, 18% had an investigation for a urinary tract infection, and 5% had an imaging study. Patients could choose more than one option; there was no option for “did not seek treatment,” so compiled data represent only the portion of patients who sought treatment.

**Figure 1:**
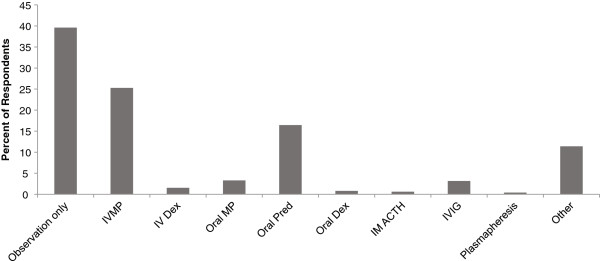
**Distribution of relapse treatments among patients with a relapse (n=4482); patients could chose more than 1 option.** ACTH, adrenocorticotropic hormone; Dex, dexamethasone; IM, intramuscular; IV, intravenous; IVIG, intravenous immunoglobulin; IVMP, intravenous methylprednisolone; MP, methylprednisolone; Pred, prednisone.

A total of 1775/4482 (40%) patients reported their relapse was managed with observation only. Collectively, IV and oral corticosteroids were the most frequently reported active treatments, the most common of which was IVMP (n =1133; 25% of total respondents) (Figure 1). Significant differences between males and females were noted in the observation-only (46% males, 38% females; *p *< 0.0001 for distribution of yes/no responses), IVMP (21%, 26%; *p *= 0.0017), and oral prednisone groups (12%, 17%; *p *= 0.0003). The age distribution of patients treated with IV steroids differed significantly from the age distribution of those not treated with IV steroids (*p *< 0.0001). Treatment with both IV and oral steroids was more likely than not at younger ages (Table 2).

**Table 2:** Number (%) of patients reporting treatment with IV or oral steroids based on age

	**IV Steroids**		**Oral steroids**	
**Age category**	**Yes**	**No**	**Yes**	**No**
20-34	113 (9.5)	172 (5.2)	65 (7.2)	220 (6.2)
35-44	332 (27.8)	683 (20.8)	240 (26.6)	775 (21.7)
45-54	484 (40.4)	1311 (39.9)	366 (40.5)	1429 (40)
55-64	226 (18.9)	901 (27.4)	192 (21.2)	935 (26.2)
65-74	35 (2.9)	179 (5.5)	37 (4.1)	177 (5)
75-90	6 (.5)	37 (1.1)	4 (0.4)	39 (1.1)
Total (n)	1196	3283	904	3575

Treatment settings varied, with 28% receiving treatment in a physician’s office, 17% in an outpatient clinic, 10% in an inpatient setting, 7% in an emergency room, 2% in an urgent care center, and 40% in home/nonclinical settings (patients could select more than 1 option). IVMP was the most common treatment in emergency room (109/316; 34%), outpatient clinic (342/766; 45%), and inpatient settings (190/427; 45%); while observation only was the most common management approach in urgent care centers (28/71; 39%) and physicians’ offices (614/1274; 48%). Observation only, IVMP, and oral prednisone were almost equally prescribed for patients in home/nonclinical settings (504 [28%], 510 [28%], and 467 [26%] of 1794, respectively).

Following their relapse (n = 4482), 68% of patients had follow-up with a physician, 20% were referred to physical therapy, 12% had a change in DMT, 7% were referred to occupational therapy, and 2% were referred to speech therapy (7% of patients indicated other responses, which included MRI, referral to other specialists or rehabilitation, medication change, urological testing, or none).

To better understand the circumstances associated with the MS relapse experience for males as compared to females, a focused analysis on how sex impacted treatment factors was performed. Males were more likely than females to be simply observed for their relapse (*p *< 0.0001), less likely than females to be treated with IVMP (*p *= 0.0017), and less likely than females to be treated with oral prednisone (*p *= 0.0003). Although the distributions of time since last relapse and number of relapses were similar for males and females, a greater proportion of males were in older age categories than females (*p *< 0.001). When experiencing a relapse, males and females utilized urgent care centers, the emergency room, and the physician’s office to the same degree. However, females were more likely to consult their doctor or nurse by phone (*p *< 0.0001). Males and females had the same likelihood of following up with a physician.

### Overall response to relapse management

**Figure 2:**
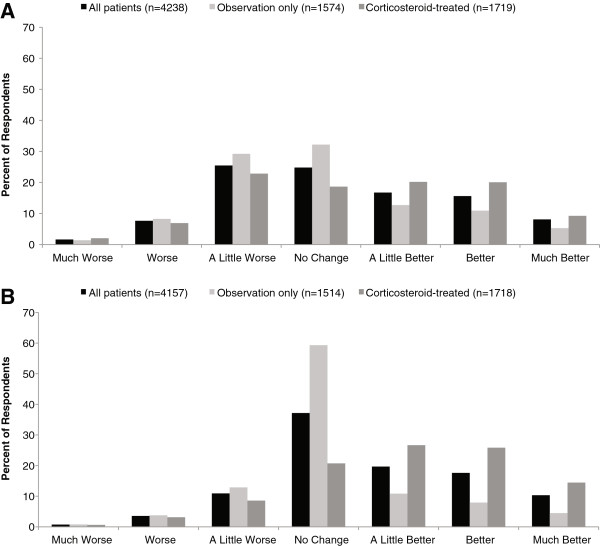
**Subjective ratings of (A) symptom improvement and (B) effect of treatment on recovery among all patients who experienced a relapse, and subgroups of interest (ie, patients whose relapses were managed with observation only or patients treated with corticosteroid). A**. Symptom improvement, based on responses to the following question: “As compared to the symptoms just before my most recent relapse, my overall MS symptoms 1 month after the relapse treatment were…” **B**. Effect of treatment on recovery, based on responses to the following question: “As a result of my treatment, my recovery was…”.

In response to the question regarding symptoms at 1 month after relapse treatment, for the overall population (n = 4238), 35% of patients reported worse symptoms, 25% reported no change, and 40% reported symptom improvement (Figure 2A). In response to the question regarding effect of treatment (including observation) on recovery, for the overall population (n = 4157), 15% of patients reported that treatment made their recovery worse, 37% reported no change, and 48% reported treatment made recovery better (Figure 2B).

The distribution of responses (worse, no change, or better) reflected less favorable outcomes for males compared with females for patient-reported symptom improvement (males: 43%, 25%, 32%; females: 33%, 25%, 43%; *p *< 0.0001) and treatment success (males: 19%, 41%, 41%; females: 14%, 36%, 49%; *p *= 0.0001). The time since last relapse trended toward being longer among those who rated their symptoms as better and among those who rated their treatment efficacy as better, than among those who indicated no change or worse for each question (data not shown).

A relationship between treatment location and symptoms ratings was seen for emergency room, urgent care center, outpatient clinic, and inpatient services, and between location and treatment efficacy in all settings (ie, the distribution of response for any given treatment setting was statistically significantly different compared with the distribution among all patients not treated in that setting) (Table 3).

**Table 3:** Number (%) of patients reporting worse, no change, or improved outcomes based on treatment setting

**Setting**	**Symptom improvement**					**Effect of treatment on recovery**				
	**n**	**Worse**	**No change**	**Improved**	***p*-value**	**n**	**Worse**	**No change**	**Improved**	***p*-value**
Home/nonclinical site	1762	614 (35)	418 (24)	730 (41)	0.3427	1755	255 (15)	569 (32)	931 (53)	<0.0001
Physician’s office	1252	452 (36)	306 (24)	494 (39)	0.4855	1238	196 (16)	494 (40)	548 (44)	0.0173
Hospital outpatient	754	258 (34)	139 (18)	357 (47)	<0.0001	745	108 (15)	192 (26)	445 (60)	<0.0001
Hospital inpatient	422	178 (42)	62 (15)	182 (43)	<0.0001	417	102 (24)	85 (20)	230 (55)	<0.0001
Emergency room	303	134 (44)	50 (17)	119 (39)	0.0002	292	84 (29)	58 (20)	150 (51)	<0.0001
Urgent care center	70	34 (49)	10 (14)	26 (37)	0.0264	62	18 (29)	17 (27)	27 (44)	0.0078

*p*-values are for effect of location on outcome from Pearson’s chi-square test; specifically, the *p*-value for each row compares that row to the distribution in all subjects not in that row.

For each treatment subgroup (ie, observation only, IVMP, oral corticosteroids, and other), there was an effect on symptom improvement and on treatment efficacy (ie, the distribution of response for any given intervention was significantly different compared with the distribution among all patients not receiving that intervention) (Table 4).

**Table 4:** Number (%) of patients reporting worse, no change, or improved outcomes by treatment

**Treatment**	**Symptom improvement**					**Effect of treatment on recovery**				
	**n**	**Worse**	**No change**	**Improved**	***p*-value**	**n**	**Worse**	**No change**	**Improved**	***p*-value**
Observation only	1574	612 (39)	507 (32)	455 (29)	<0.0001	1514	264 (17)	898 (59)	352 (23)	<0.0001
IVMP	1123	363 (32)	192 (17)	568 (51)	<0.0001	1122	145 (13)	190 (17)	787 (70)	<0.0001
Oral corticosteroids	895	301 (34)	177 (20)	417 (47)	<0.0001	894	116 (13)	224 (25)	554 (62)	<0.0001
Other	745	237 (32)	160 (21)	348 (47)	0.0005	740	114 (15)	196 (26)	430 (58)	<0.0001

IVMP, intravenous methylprednisolone.

Oral corticosteroids include oral methylprednisolone, oral dexamethasone, and oral prednisone. Other includes intramuscular adrenocorticotropic hormone, intravenous immunoglobulin, plasmapheresis, and other responses.

*p*-values are for effect of treatment on outcome from Pearson’s chi-square test; specifically, the *p*-value for each row compares that row to the distribution in all subjects not in that row.

### Outcomes among patients whose relapses were managed with corticosteroids or observation only

**Figure 3:**
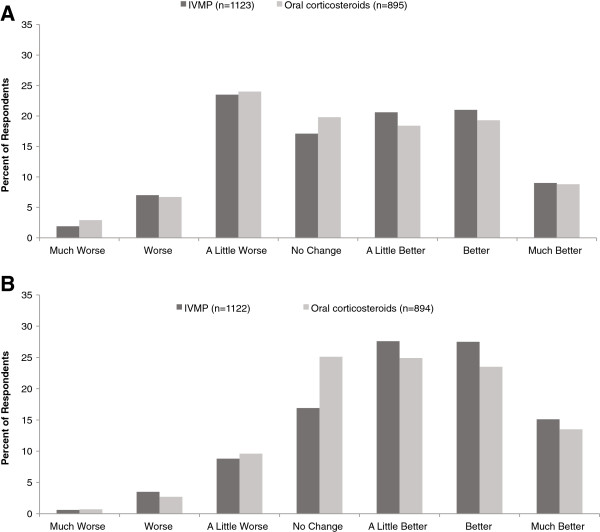
**Subjective ratings of (A) symptom improvement and (B) effect of treatment on recovery among corticosteroid-treated patients.** IVMP, intravenous methylprednisolone.

Because corticosteroids (as a whole) were the most common active treatment, we further examined outcomes among these patients. Responses for the overall corticosteroid subgroup (ie, patients treated with any IV or oral steroid) are shown in Figure 2A and 2B. We further analyzed subgroups treated with IVMP or oral corticosteroids (given that IVMP was the most common IV corticosteroid treatment reported here and in clinical trials, we excluded the small numbers of patients receiving other IV corticosteroids to strengthen our ability to draw conclusions from this analysis); statistical comparisons between IVMP-treated and oral corticosteroid-treated groups were not performed. Patient-reported symptom ratings were similar with IVMP and oral corticosteroids (Figure 3A). Responses to the question regarding treatment effects had a similar trend for IVMP and oral corticosteroids, but there were more positive responses among IVMP-treated patients (Figure 3B). Notably, approximately one-third of patients in each group (32% IVMP, 34% oral corticosteroids) reported that their symptoms were worse than prior to the relapse. Likewise, 30% of IVMP-treated and 38% of oral corticosteroid-treated patients reported that treatment resulted in no change or worsened recovery. Among IVMP-treated and oral corticosteroid-treated patients, females reported better outcomes than males. For example, among IVMP-treated patients, symptom ratings of worse, no change, or better were 30%, 17%, 53% for females (n = 943) and were 43%, 19%, 38% for males (n =180), and treatment effect ratings were 13%, 16%, 71% for females (n = 942) and 13%, 21%, 66% for males (n =180). Among oral corticosteroid-treated patients, symptom ratings of worse, no change, or better were 32%, 20%, 48% for females (n = 759) compared with 41%, 21%, 38% for males (n =136), and treatment effect ratings were 13%, 24%, 63% for females (n =758) and 13%, 29%, 59% for males (n =136).

Outcomes among patients whose relapses were managed with observation only were generally poorer than outcomes with treatment. Most (71%) respondents in this subgroup reported that their symptoms were not changed or worse than before their relapse (Figure 2A), and 77% reported that their treatment had no effect or made their recovery worse (Figure 2B). As with other treatments, males tended to have less symptom improvement (n = 348; 48% worse, 28% no change, 24% better) than females (n = 1226; 36% worse, 34% no change, 30% better) and less treatment efficacy (n = 342; 22% worse, 57% no change, 21% better) than females (n = 1172; 16% worse, 60% no change, 24% better).

### Factors affecting response to treatment

Factors associated with patient-perceived symptoms at 1 month after treatment included sex, number of relapses, and time since last relapse. The odds of patient-reported symptoms being “not worse” were lower for males than females (odds ratio [OR], 0.63; 95% confidence interval [CI], 0.54-0.75) and for patients with 5 to 9 relapses (OR, 0.75; 95% CI, 0.64-0.89) or ≥10 relapses (OR, 0.68; 95% CI, 0.58-0.80), compared with those with 1 to 4 relapses, and were higher with a greater number of years since the last relapse (OR, 1.21; 95% CI, 1.11-1.32) (Table 5).

**Table 5:** Summary of logistic regression results

				**95% Confidence interval**		
**Outcome**	**Model variables**	**Beta coefficient**	**Odds ratio**	**Lower limit**	**Upper limit**	***p-*value**
Symptoms	Sex	−0.455	0.63	0.54	0.75	<0.001
	Number of Relapses (5–9)	−0.287	0.75	0.64	0.89	<0.001
	Number of Relapses (10+)	−0.387	0.68	0.58	0.80	<0.001
	Years Since Most Recent Relapse	0.192	1.21	1.11	1.32	<0.001
Recovery	Sex	−0.211	0.81	0.68	0.96	0.017
	Years Since Most Recent Relapse	0.185	1.20	1.11	1.31	<0.001
	Treatment With IV Steroids	1.306	3.69	3.15	4.33	<0.001
	Treament With Oral Steroids	0.868	2.38	1.94	2.92	<0.001
	Other Treatment	1.095	2.99	2.08	4.29	<0.001
	Treated at Home or Other Nonclinical Site	1.206	3.34	2.49	4.47	<0.001
	Treated at Clinical Site	1.14	3.13	2.36	4.14	<0.001

Factors associated with patient-reported treatment success (ie, responses indicating treatment made recovery “better” vs “not better”) were sex, time since last relapse, treatment, and treatment setting. The odds of treatment success were lower for males than females (OR, 0.81; 95% CI, 0.68-0.96) and higher with a greater number of years since the last relapse (OR, 1.20; 95% CI, 1.11-1.31), and with home/nonclinical site treatment (OR, 3.34; 95% CI, 2.49-4.47) or clinical site treatment (OR, 3.13; 95% CI, 2.36-4.14) compared with patients whose treatment location was unspecified. All treatment groups had greater odds of treatment success compared with observation only: IV corticosteroids (OR, 3.69; 95% CI, 3.15-4.33), oral corticosteroids (OR, 2.38; 95% CI, 1.94-2.92), and other (OR, 2.99; 95% CI, 2.08-4.29) (Table 5).

## Discussion

This retrospective analysis of data from the NARCOMS Registry demonstrates that treating relapses results in better patient-reported outcomes compared with observation only. However, one-third of patients treated with the most common treatment, IVMP, reported suboptimal outcomes in symptom improvement and the effect of treatment on their recovery. Although some clinicians may recognize this observation in their practice, physician and patient perceptions may differ, and the extent to which patients report suboptimal outcomes has not been quantified.

It is challenging to compare our findings with other studies of corticosteroid treatment for MS relapses. Clinical trials of corticosteroid treatment for relapses have established variable rates of recovery, which may be due to differences in factors such as how recovery is defined and the timing of recovery evaluation [17]. In a Cochrane review of corticosteroids, standardized outcomes were used to evaluate combined data from 3 studies [18]. Improvement in EDSS scores was documented 4 weeks after treatment in 45% of patients on oral corticosteroids and 59% of patients on IV corticosteroids (consistent with the percentage of corticosteroid-treated patients reporting improved symptoms at 1 month after treatment in this analysis of NARCOMS data); however, improvement rates in the individual studies ranged from 19% to 100%, suggesting substantial clinical and individual variability in the relapse experience.

Another consideration is that most studies have used standardized clinician-rated outcome measures, whereas we have evaluated patient-reported outcomes. Although several studies have examined changes in, and correlations among, clinician-rated measures (eg, the EDSS and Multiple Sclerosis Functional Composite) and patient-rated HRQoL measures for evaluating response to relapse treatment [19-21], data on patient-perceived recovery are limited, and agreement between clinician-rated and patient-reported outcomes can be poor. One study evaluated outcomes among patients treated with IV corticosteroids after relapse and included a 5-point Likert-type scale for patient-reported recovery; 70% of patients reported improvement (ie, a rating of “somewhat better” or “much better” than 2 months ago) over an 8-week follow-up period [22]. This finding is consistent with the 71% of IV corticosteroid-treated patients reporting that treatment improved their recovery in our analysis. Hoogervorst and colleagues reported a slightly higher rate of patient-reported improvement (78%; 21/27 patients with RRMS) after treatment with IVMP [23].

In our analysis, several factors affected relapse outcomes (regardless of treatment), including the number of previous relapses, time since last relapse, and sex; we also observed that corticosteroid treatment was associated with improvement less frequently in males than in females. Others have observed a greater frequency of improvement in EDSS scores among females (81%) compared with males (65%) at 3 months (OR, 2.33; 95% CI, 1.03–2.56; Mantel-Haenszel *p *= 0.024) [24], which is consistent with the lower odds of improvement for males overall and the lower percentages of improvement in corticosteroid-treated males in this analysis. However, Hirst and colleagues [4] reported no effect of sex on recovery in corticosteroid-treated patients.

In the NARCOMS Registry, 40% of the most recent relapses were not treated; they were only observed. The reasons for this finding are unclear, since data were not collected regarding the reasons for treatment initiation, specific relapse symptoms, severity of symptoms, or the extent of disability or impairment of HRQoL associated with the relapse. Thus, it is possible that many relapses did not cause enough disability to warrant treatment. In the study by Hirst and colleagues, only 30.5% of patients had relapses that required treatment [4], suggesting that our finding is not unusual. Alternatively, it is possible that in some instances patients were reluctant to pursue interventional therapy or to report relapses to their health care provider but were more likely to report relapse occurrence in the survey. However, it is also possible that the high number of untreated relapses reflects patient dissatisfaction with corticosteroid relapse therapy based on their previous experiences. That is, nearly one-third of corticosteroid-treated patients felt that the treatment made their symptoms worse, suggesting that they experienced discomfort related to adverse events; an additional 15% to 20% reported no change in symptoms. As such, some patients may have previously experienced discomfort with corticosteroid-related adverse events or had a suboptimal response and, therefore, opted not to receive treatment for the most recent relapse. On a broader level, there may be an unrecognized propensity for dissatisfaction with corticosteroid treatment as a whole.

Regardless of the reason for the substantial proportion of untreated relapses, we cannot exclude the possibility that findings with respect to the extent of symptom improvement and effects of treatment on recovery might have differed had these relapses actually been treated with corticosteroids. Treatment of relapses appears to improve patients’ perceptions of recovery in the short term, based on this large sample of survey respondents. Although we are unable to address long-term patient-reported outcomes with these data, treating relapses also may be important in terms of effects on overall MS outcomes and HRQoL. For example, Healy and colleagues reported that relapses were associated with not only reduced HRQoL at the time of a relapse, but also an overall decline in patient-rated outcomes over a period of 1 year, compared with patients in remission [25]. Acute relapse treatment with corticosteroids (or ACTH) is not considered to be disease modifying and has not been shown to result in long-term benefits [26]. However, evidence that acute treatment shortens the time to recovery [18] suggests that corticosteroid interventions help to more quickly resolve the pathophysiological mechanisms responsible for clinical relapses. There is evidence that inflammation and demyelination are associated with processes that contribute to long-term disability (ie, axonal damage or loss), that these processes begin early in the course of MS, and that the extent of damage increases with the severity of inflammatory injury [27-29]; if treatment of relapses reduces the extent of damage by helping to more quickly resolve inflammatory processes, there may be long-term benefits that are not yet recognized. Thus, the shortening of relapses and hastening of resolution of symptoms and functional impairment may be associated with greater overall HRQoL, and effects on the underlying pathophysiological processes may ultimately benefit patients in the long term.

Limitations of this analysis include the retrospective nature of data collection and the reliance on patient recall. It is unlikely that patients were prospectively documenting their relapses and treatment responses. Given the mean time since last relapse of 11 months, most patients had to provide information based on their recollection of the relapse several months after the fact. In addition, information on dosage regimen was not reported consistently by respondents, and therefore we were unable to include dose as an independent variable in order to evaluate the effect of dosage regimen on outcomes. It is possible, for example, that the response in males was worse because they did not have an optimal dosage regimen or actually require a different regimen. Also, the questionnaire did not collect information related to the tolerability of relapse treatments; thus, we are unable to comment on whether and the extent to which adverse events contributed to patients’ perceptions of symptom improvement and treatment effects. Selection bias is also possible given that only 63.4% of participants returned the questionnaire; it is unknown whether relapse history or treatment responses differed between responders and non-responders. Finally, because patient-reported outcomes frequently are not standardized, comparisons between studies can be difficult. These limitations are offset by the large number of patients included in the analysis. Although there is variability in some of the results, the overall perceptions of patients inform clinical care.

## Conclusions

Taken together, these data indicate that, to mitigate the severity of relapse-related pathophysiology and improve patient HRQoL, it may be necessary to consider alternatives to corticosteroids in RRMS patients who do not adequately respond to such treatment for a relapse. Clinical and objective measures evaluating the therapeutic response, side-effect profile, and patient HRQoL following relapse treatment regimens are warranted and may lead to improved relapse management.

## Abbreviations

ACTH: Adrenocorticotropin hormone; CI: Confidence interval; CMSC: Consortium of multiple sclerosis centers; DMT: Disease-modifying therapies; EDSS: Expanded disability status scale; HRQoL: Health-related quality-of-life; IG: Immunoglobulin; IM: Intramuscular; IVMP: Intravenous methylprednisolone; MRI: Magnetic resonance imaging; MS: Multiple sclerosis; NARCOMS Registry: North American research committee on multiple sclerosis registry; OR: Odds ratio; RRMS: Relapsing-remitting multiple sclerosis.

## Competing interests

MN is an employee of Questcor Pharmaceuticals. RAM has received funding for clinical trials from Sanofi-Aventis and has received research support from the Canadian Institutes of Health Research, Public Health Agency of Canada, MS Society of Canada, MS Scientific Foundation, Manitoba Health Research Council, and the Health Sciences Centre (HSC) Foundation.

## Authors’ contributions

MN and RAM contributed substantially and were involved in data analysis/interpretation and in drafting or critically revising the manuscript. The authors outlined the manuscript, reviewed all drafts of the manuscript, and gave approval for submission. Both authors read and approved the final manuscript.

## Pre-publication history

The pre-publication history for this paper can be accessed here:

http://www.biomedcentral.com/1471-2377/13/119/prepub
